# Emergence of a genetically distinct cluster of influenza A(H3N2) viruses within subclade J.2.2 associated with hospitalization during the 2024–2025 season in Auvergne–Rhône–Alpes, France

**DOI:** 10.1099/mgen.0.001810

**Published:** 2026-09-22

**Authors:** Lama Koumaty, Stéphanie Dan, Allan De Clercq, Grégory Destras, Hadrien Regue, Antoine Oblette, Adrien Le Meur, Alexandre Gaymard, Vanessa Escuret, Maude Bouscambert-Duchamp, Emmanuel Chanard, Margaux Fabre, Vincent Vieillefond, Benoit Visseaux, Antonin Bal, Laurence Josset

**Affiliations:** 1 Centre National de Référence des virus des infections respiratoires, Institut des Agents Infectieux, Hospices Civils de Lyon, 69004, Lyon, France; 2 GenEPII Sequencing Platform, Institut des Agents Infectieux, Hospices Civils de Lyon, 69004, Lyon, France; 3 CIRI, Centre International de Recherche en Infectiologie, Team VirPath, Univ Lyon, Inserm, U1111, Université Claude Bernard Lyon 1, CNRS, UMR5308, ENS de Lyon, 69007, Lyon, France; 4 Center of Excellence in Respiratory Pathogens (CERP), Hospices Civils de Lyon (HCL), Lyon, France; 5 Service de Microbiologie, Cerballiance Auvergne-Rhône-Alpes, Lyon, France; 6 UNILIANS-BIOGROUP, Décines-Charpieu, France; 7 BPO-BIOEPINE-Biogroup, Levallois-Perret, France; 8 Laboratoire Cerba, Département d'infectiologie, Laboratoire Cerba, Cerba Healthcare, Frépillon, France

**Keywords:** genomic surveillance, influenza, public health, severity, whole-genome sequencing

## Abstract

During the 2024–2025 influenza season in Auvergne–Rhône–Alpes, France, a genetically distinct cluster of A(H3N2) viruses within subclade J.2.2 was found to be significantly overrepresented in hospitalized patients compared with community patients. Circulating mainly in November and December 2024, this genetically distinct cluster harboured HA1:G62R and S145N mutations, both linked to immune escape, as well as P239Q, located near key antigenic sites and warranting further investigation. The findings highlight the importance of monitoring influenza genome diversity in both hospital and community settings to identify emerging genetically distinct clusters with potential for increased severity.

**Impact Statement**

Genomic surveillance during the 2024–2025 influenza season in Auvergne–Rhône–Alpes, France, identified a newly emerging genetically distinct cluster within A(H3N2) subclade J.2.2 (J.2.2-B) that was associated with higher odds of hospitalization. By combining whole-genome sequencing with clinical data, the study highlights how high-resolution genomic monitoring can detect clusters linked to increased disease severity. The proposed analytical framework is adaptable to future influenza seasons and other respiratory viruses, providing actionable evidence for clinicians, laboratories and public health authorities.

## Data Summary

The influenza virus sequences generated in this study have been deposited in GISAID and GenBank. Complete genomes used for the concatenated whole-genome phylogenetic analysis are available through GISAID (EPI_SET_260619vs; DOI: https://doi.org/10.55876/gis8.260619vs). A summary of the GISAID EPI_SET is provided in Appendix S1. GenBank accession numbers for available segments, together with the corresponding GISAID accession numbers, isolated metadata and information on genome completeness and inclusion in the whole-genome phylogenetic analysis, are provided in the Table S2 Excel file.

## Introduction

The 2024–2025 influenza season in France was marked by unusually high clinical impact, with early onset, prolonged circulation and elevated mortality. The epidemic lasted 12 weeks, resulting in 29,000 hospitalizations and 14,100 excess all-cause deaths, the highest mortality rate since 2020 [1]. Nationally, A(H1N1) pdm09 (35%), B/Victoria (33%) and A(H3N2) (26%) co-circulated during the 2024–2025 season [1]. This study examined whether certain genetically distinct clusters were disproportionately identified in samples from hospital-based laboratories at Hospices Civils de Lyon (HCL) compared with community-based laboratories within the Réseau des Laboratoires d’Auvergne–Rhône–Alpes (RELAB-ARA).

## Methods

### Surveillance networks

Data from two surveillance networks in the Auvergne–Rhône–Alpes (ARA) region were used: (i) the university multi-site hospital of Lyon (HCL), with around 6,000 beds, and (ii) the RELAB-ARA network [2], a consortium of public and private laboratories conducting routine influenza diagnostics in community settings.

### Clinical samples

A total of 2,616 influenza-positive respiratory samples collected between 1 September 2024 and 28 February 2025 were selected. This dataset included 1,713 samples from HCL hospital-based laboratories (from hospitalized patients and those presenting to the emergency department), for which all specimens with a cycle threshold value <28 were sequenced, and 903 samples from the RELAB-ARA community laboratory network, corresponding to ~10.5% (903 out of 8,604) of positive detections, which were randomly selected, yielding a median weekly sequencing proportion of 15.2% (interquartile range: 16.6%) (Fig. S1, available in the online Supplementary Material).

### Influenza whole-genome sequencing

RNA extraction was performed with the MGISP-960 workstation using the Easy Magnetic Beads Virus DNA/RNA Extraction kit (1000021043, MGI Tech, Marupe, Latvia). Contaminating DNA was removed using ezDNase for 5 min at 37 °C, and whole genomes were amplified using two primer sets published in [3, 4] and SuperScript III One StepReverse transcription polymerase chain reaction (RT-PCR) (11732088, Life Technologies, Saint-Aubin, France). Libraries were prepared with Illumina CovidSeq Assay (4080836–20043675, Illumina Inc, Eindhoven, Netherlands) and sequenced on NovaSeq 6000 (Illumina) at 2×100 bp.

### Bioinformatics

Reads were processed using the in-house bioinformatic pipeline seqmet (available at https://github.com/genepii/seqmet). Software versions and parameters are reported in Table S1. Reference genomes used for read alignments were EPI_ISL_200780 for A(H1N1) pdm09, EPI_ISL_129744 for A(H3N2) and EPI_ISL_219327 for B/Victoria.

### Phylogenetic analysis

Full genome coverage (defined as >95% coverage in all 8 segments) was achieved in 2,282 samples, which were used for whole-genome phylogenetic analysis. Consensus genomes were aligned with MAFFT v7.31342 (--auto) and minimally edited in AliView v1.19 [5]. Maximum likelihood (ML) trees were inferred in IQ-TREE v2.0 under GTR+G (4 discrete Γ categories) with 1,000 ultrafast bootstraps [6]. Clades followed WHO GISRS HA-based rules; non-haemagglutinin (HA) segments were assigned by clustering with the corresponding parental HA clades in segment-specific trees. All segments were aligned on the reference segments of A/Darwin/6/2021 with Nextclade (v3.21.2). Treetime (v0.11.4) [7] was used to reconstruct the ancestral nucleotides and amino acid states from the whole-genome tree using parsimony. Trees were visualized in R with ggtree v2.3.5 [8].

### Statistical analysis

The HA segment was successfully sequenced with >95% coverage in all 2,616 samples, enabling inclusion in subclade-level statistical analyses. All statistical analyses were conducted using R software (version 4.4.2) [9] with the following packages: dplyr [10], tidyr [11], ggplot2 [12], broom [13] and patchwork [14]. Comparisons were performed using chi-square or Fisher’s exact tests, as appropriate. To assess associations between hospitalization and influenza subtype or subclade, univariate and multivariate logistic regression models were constructed. Multivariate models were adjusted for age group and month of sample collection. Results were expressed as odds ratios (ORs) with corresponding 95% CIs and *P* values.

### Ethics

The study protocol received approval from the institutional review board of the HCL (Comité Scientifique et Éthique des Hospices Civils de Lyon) in June 2023 (reference 23-5039).

## Results

### Age extremes and later detection of influenza circulation in a hospital setting

Patient demographics and timing of detection were compared between the two networks (Table 1). Patients tested at the hospital (HCL) were more likely to be older than 65 years (34.7% vs. 23.9% in RELAB-ARA) or younger than 2 years (5.4% vs. 1.3% in RELAB-ARA). In addition, influenza circulation was detected earlier in the community than in the hospital, with 2% of the RELAB-ARA cases detected in September–October and 10% in November vs. <0.1% in September–October and 2% in November for HCL cases.

**Table 1.** Characteristics of the study population by clinical setting, ARA, France, September 2024–February 2025 (*n*=2,616)

**Characteristic**	**Clinical setting**				***P*** ** value**
	**HCL, *n*=1,713** **Hospitalized,** ***n*****=615** **Emergency,** ***n*****=1,098**		**RELAB-ARA, *n*=903**		
	**Number**	**%**	**Number**	**%**	
**Sex**					
Female	972	56.7	527	58.4	0.451
Male	741	43.3	376	41.6	
**Age group in years**					
**<2**	**92**	**5.4**	**12**	**1.3**	*P*<0.001
2–17	279	16.3	202	22.4	
18–64	736	43	471	52.2	
**≥65**	**595**	**34.7**	**216**	**23.9**	
**Unknown**	11	0.6	2	0.2	0.241
**Month – year of testing**					
Sep-2024	6	0	15	2	***P*<0.001**
Oct-2024	8	0	21	2	***P*<0.001**
Nov-2024	34	2	89	10	***P*<0.001**
Dec-2024	473	28	139	15	***P*<0.001**
Jan-2025	763	45	380	42	0.244
Feb-2025	429	25	259	29	0.0497

### A(H3N2) virus was associated with hospitalization

A(H3N2) viruses were significantly more frequent in hospital-derived samples (46.5%) compared to RELAB-ARA (36.1%, *P*<0.001), whereas B/Victoria was more common in community samples (30.6% vs. 21.4%, *P*<0.001) (Table 2). Subtype-specific patterns over time are shown in Fig. S2.

**Table 2.** Distribution of influenza virus subtypes, clades and subclades by clinical setting (HCL vs. RELAB-ARA), 2024–2025 season

**Characteristic**	**Clinical setting**				***P*** ** value**
	**HCL, *n*=1,713** **Hospitalized,** ***n*****=615** **Emergency,** ***n*****=1,098**		**RELAB, *n*=903**		
	**Number**	**%**	**Number**	**%**	
**Influenza subtype**					
A(H1N1)	549	32	301	33.3	0.533
A(H3N2)	**797**	**46.5**	**326**	**36.1**	***P*<0.001**
BVIC	367	21.4	276	30.6	*P*<0.001
**Subtype – clade (proportion among each subtype)**					
A(H3N2)	***N*=797**		***N*=326**		
A(H3N2) – 3C.2a1b.2a.2a.3a	1	0.1	0	0	1.000
A(H3N2) – 3C.2a1b.2a.2a.3a.1	796	99.9	326	100	1.000
A(H1N1)pdm09	***N*=549**		***N*=301**		
A(H1N1)pdm09 – 6B.1A.5a.2a.1	76	13.8	32	10.6	0.216
A(H1N1)pdm09 – 6B.1A.5a.2a	473	86.2	269	89.4	0.216
BVIC – V1A.3a.2	367	100	276	100	1.000
**Subtype – subclade**					
A(H1N1)	***N*=549**		***N*=301**		
A(H1N1)-C.1.9	86	15.7	50	16.6	0.793
A(H1N1)-C.1.9.1	22	4.0	7	2.3	0.274
A(H1N1)-C.1.9.2	1	0.2	0	0.0	1.000
A(H1N1)-C.1.9.3	355	64.7	208	69.1	0.217
A(H1N1)-C.1.9.4	9	1.6	4	1.3	1.000
A(H1N1)-**D**	44	8.0	12	4.0	0.034
A(H1N1)-D.1	2	0.4	1	0.3	1.000
A(H1N1)-D.3	20	3.6	10	3.3	0.962
A(H1N1)-D.5	10	1.8	9	3.0	0.390
**A(H3N2)**	***N*=797**		***N*=326**		
A(H3N2)-G.1.3.1	1	0.1	0	0.0	1.00
A(H3N2)-J.2	16	2	11	3.4	0.253
A(H3N2)-**J.2.1**	11	1.4	13	4.0	0.012
**A(H3N2)-J.2.2**	**206**	**25.8**	**47**	**14.4**	**<0.001**
A(H3N2)-**J.2.a**	**367**	**46.0**	**176**	**54.0**	**0.019**
A(H3N2)-J.2.b	7	0.9	6	1.8	0.289
A(H3N2)-J.2.c	183	23.0	68	20.9	0.491
A(H3N2)-J.2.d	6	0.8	5	1.5	0.383

PeD.

**Fig. 1.**
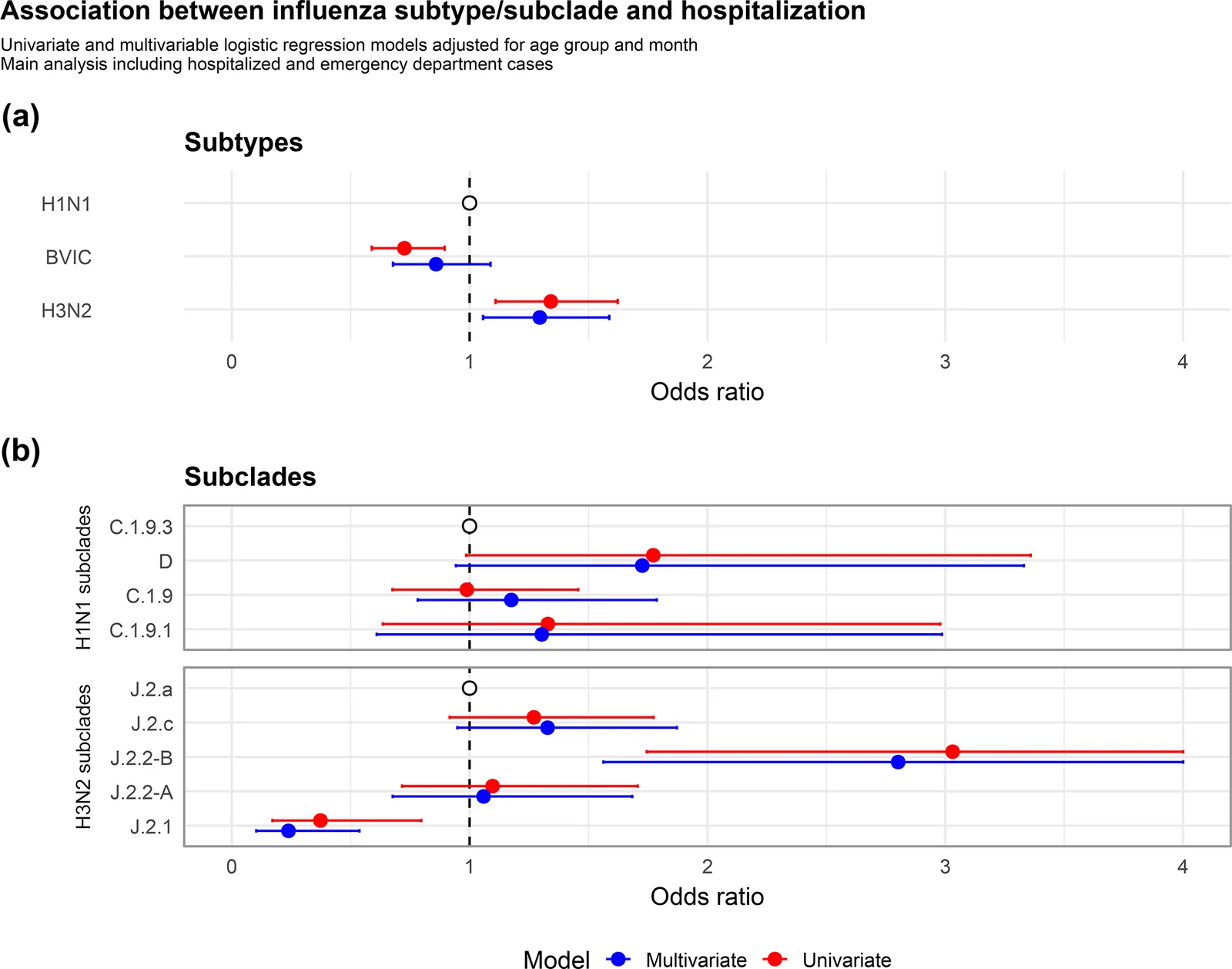
Association between influenza subtype/subclade and hospitalization, ARA, 2024–2025. Forest plot of ORs and 95% CIs for hospitalization according to influenza subtype and subclade. The primary analysis included both hospitalized patients and emergency department cases from HCL. Panel (a) shows subtype-level analyses, and panel (b) shows subclade-level analyses. Red points and lines represent univariate logistic regression models, and blue points and lines represent multivariable logistic regression models adjusted for age group (reference: 18–64 years) and month of diagnosis (reference: September 2024). The vertical dashed line indicates OR=1 (no association with hospitalization). Open circles represent the reference categories within each grouping, fixed at OR=1 (H1N1 among subtypes, C.1.9.3 among A(H1N1)pdm09 subclades and J.2.a among A(H3N2) subclades). Filled circles indicate estimated ORs for each subtype or subclade. H1N1 and H3N2 subclades are displayed separately.

To quantify the association between viral subtype and clinical setting, logistic regression was performed. Given differing age distributions (Fig. S3) and temporal variation (Figs S2 and S4), models were adjusted for age group and month of collection. A(H3N2) viruses had higher odds of hospital-based detection than A(H1N1) pdm09 (aOR=1.29; 95% CI: 1.05–1.59), while B/Victoria showed no significant association in multivariate analysis (Fig. 1a).

### No association of A(H1N1) and B subclades with hospitalization

Whole-genome phylogenies of A(H1N1) pdm09 (*n*=697; Fig. S5) and B/Victoria (*n*=595; Fig. S6) showed similarly intermingled hospital and community samples within co-circulating subclades C.1.9.* and D.* for A(H1N1) pdm09 and subclades C.3, C.5.1, C.5.6 and C.5.7 for the V1A.3a.2 lineage of B/Victoria. While the A(H1N1) D subclade was overrepresented in HCL patients (Table 2), there was no significant association with hospitalization after covariable adjustment (Fig. 1b).

### Identification of a hospital-enriched A(H3N2) subclade

Among A(H3N2) viruses (*n*=1,123), subclade J.2.2 was significantly overrepresented in HCL patients (25.8% vs. 14.4% in RELAB-ARA, *P*<0.001) (Table 2).

**Fig. 2.**
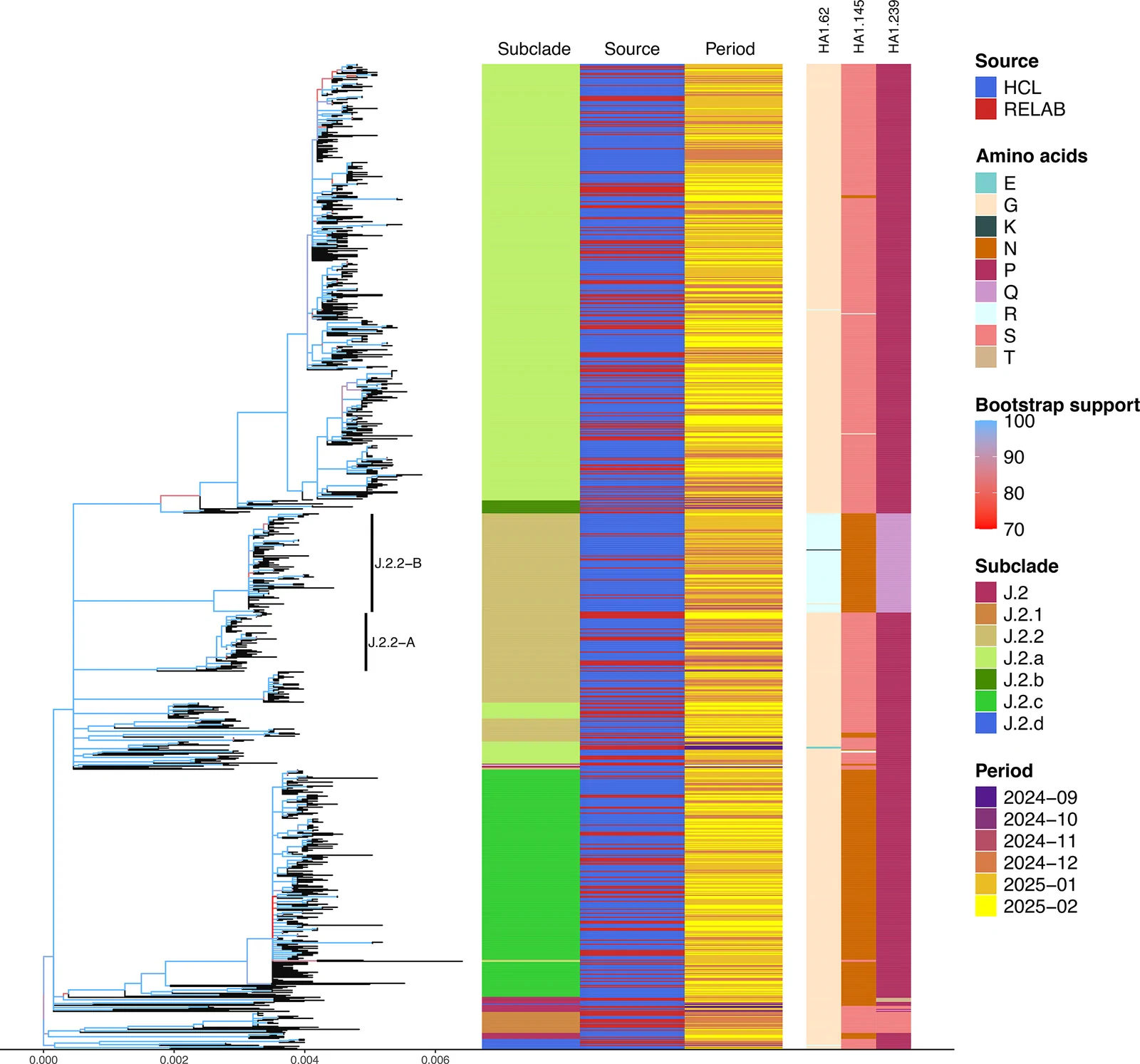
Whole-genome ML phylogenetic analysis of influenza A(H3N2) viruses (*n*=988) from ARA, France, September 2024–February 2025. ML tree inferred from 988 complete A(H3N2) genomes generated from influenza retrospective surveillance samples processed by the National Reference Center for Respiratory Viruses of the HCL. The dataset includes samples from hospitalized patients and from the RELAB network, a group of community-based laboratories in the ARA region, collected between September 2024 and February 2025. Branch lengths reflect the number of nucleotide substitutions per site. Ultrafast bootstrap support values are appended to the internal nodes. The tree was rooted using WHO reference/vaccine strains. Amino acids associated with the J.2.2-B cluster were identified by parsimony.

To better resolve the genetic diversity within subclades, phylogenetic analysis of 988 A(H3N2) whole-genome sequences was carried out (Fig. 2). Subclade J.2.2 showed clear internal diversification. By applying TreeCluster in single-linkage mode with a genetic distance cutoff of 0.005 substitutions per site on the concatenated full-genome alignment, two distinct clusters within J.2.2 were identified and designated in this study as J.2.2-A (*n*=114) and J.2.2-B (*n*=102), in addition to 37 J.2.2 sequences that were not clustered. This classification highlights the genetic diversity emerging within a single subclade over the course of the season [15]. Seven amino acid changes were associated with J.2.2-B (five in HA, one in NA and one in PB2) (Fig. S7). Three amino acids in HA1 were exclusive to J.2.2-B among the J.2.2 subclade: G62R, S145N and P239Q (reference: A/Darwin/6/2021). J.2.2-A was characterized by HA1: T65K. Notably, S145N occurs within antigenic site A of the H3 HA [16]. No evidence of an internal segment reassortment event was found at the origin of J.2.2-B.

Interestingly, J.2.2-B was more predominantly detected in HCL samples (*n*=92/797, 11.5%) than in RELAB-ARA (*n*=10/326, 3.1%), while J.2.2-A had a balanced distribution. After adjusting for covariables, J.2.2-B had the highest adjusted odds of hospitalization vs. J.2.a (aOR=2.72; 95% CI: 1,51–5.25) (Fig. 1). J.2.2-B association with hospital detection persisted in a sensitivity analysis restricted to hospitalized inpatients, excluding samples collected in the emergency department (Fig. S8). This association remained significant after adjusting for confounders.

J.2.2-B circulated primarily from November to December 2024, peaking in week 52 (Fig. S9). It was detected across different age groups, including children under 17 years and adults over 65 years (Fig. S10) and within different hospital units, excluding a nosocomial transmission. After December, it was replaced by J.2.c and J.2.d.

To evaluate the worldwide circulation of this cluster, all 19,440 A(H3N2) sequences available on GISAID and collected between 1 September 2024 and 28 February 2025 were analysed. Among the 2,696 J.2.2 sequences, there were 178 sequences characterized by the haplotype G62R-S145N-P239Q, including 130 from France, 24 from the USA, 12 from Oman, 6 from the United Arab Emirates, 5 from Europe and 1 from Japan. This demonstrates limited worldwide circulation of this cluster, with the majority of cases being detected in France.

The adjacent heat maps summarize (from left to right) the following: subclade assignment, the sample source (RELAB or HCL), collection month and the amino acids associated with the J.2.2-B cluster.

## Discussion and conclusion

The 2024–2025 influenza season resulted in a significant hospitalization burden across Europe [17]. When comparing a community-based network to hospitalized patients, a genetically distinct cluster of A(H3N2) viruses within subclade J.2.2 ‘J.2.2-B’ was found to be enriched among hospitalized patients. This finding was made possible by the high number of sequences generated at HCL (*n*=1,713), which allowed for high-resolution detection of circulating clusters. This cluster ‘J.2.2-B’ was characterized by three defining HA1 substitutions: G62R, S145N and P239Q. Among these HA1 residues, residue 145 has been clearly mapped within an antigenic site and strongly associated with an antigenic drift and immune escape [18], suggesting a potential link between viral genetic features and antigenic evolution that may have contributed to the observed epidemiological pattern. In contrast, residues 62, 65 and 239 are not part of the canonical H3 antigenic sites (A–E), although substitutions at these positions may influence local HA structure or viral fitness. According to WHO and ECDC reports, A(H3N2) viruses of subclade J.2.2 circulated at low levels globally during the 2024–2025 season, with no clinical severity data reported for this subclade [17, 19]. The Norwegian interim influenza report also described cocirculation of A(H3N2) subclades J.2 and J.2.2 and noted additional HA1 changes (T65K, S145N) within J.2.2 but did not evaluate subclade enrichment among hospitalized patients or severity outcomes [20]. Analysis of GISAID data points to limited global spread of the J.2.2-B cluster. The limited detection of this cluster in other countries, however, might be due to either lower sequencing intensity across national surveillance systems or low circulation of the cluster. France is one of the major contributors to regional genomic surveillance, as evidenced by the 5,621 A(H3N2) sequences from Europe that contain at least both HA and NA segments, of which 2,421 (43%) are from France, according to GISAID. The large volume of sequences generated at HCL alone likely increased our ability to detect this subclade, underscoring the role of sequencing coverage in identifying emerging clusters.

A(H3N2) infections were significantly associated with hospitalization than A(H1N1), even after adjusting for age and timing, highlighting a potential intrinsic severity. While A(H3N2) is often associated with higher hospital burden [21], recent findings from Sumner *et al.* suggest that A(H1N1) pdm09 was more likely to cause ICU admission, mechanical ventilation, ECMO and death than A(H3N2) in the USA between 2010 and 2019 [22]. These differences may reflect variations in study design (e.g. hospital-only vs. mixed populations), circulating strains, healthcare systems or population immunity. Previous studies have identified specific clusters of A(H3N2) associated with severity: in the USA (2017–2018), a reassortant A(H3N2) strain showed higher clinical severity scores [23]; and in Belgium (2016–2017), hospitalization was more frequent in clade 3C.2a3 and A(H3N2) reassortant viruses [24].

The epidemiology of A(H3N2) is strongly influenced by its rapid antigenic evolution, which contributes to vaccine mismatch, reduced population immunity and increased susceptibility to infection. Genomic surveillance data support a role for antigenic mismatch in increased circulation of A(H3N2) viruses. In Italy during the 2021–2022 season, A(H3N2) viruses belonging to clade 3C.2a1b.2a.2 exhibited antigenic site B substitutions, notably H156S and Y159N, raising concerns over immune evasion and vaccine mismatch [25]. In the 2024/25 Canadian SPSN study, most A(H3N2) viruses belonged to clade 2 a.3a.1, primarily subclade J.2. These viruses were characterized by the substitutions N122D and K276E, while some additionally carried substitutions within antigenic site A, including S145N or T135K [16].

While these antigenic changes may increase the number of susceptible individuals and overall virus circulation through reduced population immunity, they do not necessarily confer increased intrinsic virulence. Therefore, the association observed between the genetically distinct J.2.2-B cluster and hospitalization in our study should not be interpreted as evidence of increased pathogenicity. Rather, this association may reflect a combination of reduced population immunity and host-related factors that were not available in our dataset, including vaccination status, prior immunity, comorbidities and reasons for hospitalization.

The apparent regional restriction of the J.2.2-B cluster may also reflect several non-mutually exclusive factors, including founder effects, localized transmission dynamics, population immunity patterns and differences in geographic sampling intensity. Although the relatively high sequencing coverage achieved in our study likely increased our ability to detect this cluster, the available data do not allow the relative contribution of these factors to be determined. Further studies integrating phylogeographic, epidemiological, immunological and virological data are needed to better understand both the regional distribution of J.2.2-B and the mechanisms underlying its association with hospitalization.

This study has several limitations. Most notably, access to clinical metadata was lacking for both hospitalized (HCL) and community (RELAB) cases. This includes key information such as comorbidities, reasons for hospitalization [e.g. severe acute respiratory infection (SARI)] and vaccination status. The absence of vaccination data prevented us from evaluating whether vaccine coverage influenced the severity associated with specific influenza subtypes or subclades. In addition, it was not possible to track whether RELAB patients were subsequently hospitalized, or whether patients initially tested in the emergency room were later admitted. Another limitation is sequencing coverage. While sequencing was nearly complete for HCL cases, it only covered ~10.5% of RELAB-ARA cases, which may affect the representativeness of community samples.

Despite these limitations, subclade J.2.2-B remained significantly associated with hospitalization after covariable adjustment. These findings highlight the importance of strengthening genomic surveillance systems and integrating them with thorough clinical data to better inform vaccine strain selection and public health decision-making.

## Supplementary material

10.1099/mgen.0.001810 Supplementary Material 1.

10.1099/mgen.0.001810 Supplementary Material 2.

10.1099/mgen.0.001810 Table S1.

## Abbreviations

ARA: Auvergne–Rhône–Alpes
HA: haemagglutinin
HCL: Hospices Civils de Lyon
ML: maximum likelihood
ORs: odds ratios
RELAB-ARA: Réseau des Laboratoires d’Auvergne–Rhône–Alpes
RT-PCR: Reverse transcription polymerase chain reaction
