# Supplementary material for: Emergence of a genetically distinct cluster of influenza A(H3N2) viruses within subclade J.2.2 associated with hospitalization during the 2024–2025 season in Auvergne–Rhône–Alpes, France
Source: Microb Genom. 2026 Sep 22;12(9):001810. doi: 10.1099/mgen.0.001810 (PMC13597097; doi:10.1099/mgen.0.001810)
Supplement: Supplementary file 1 [file mgen-12-01810-s001.pdf]

Supplementary Figures

Supplementary Figure S1. Weekly Counts and Proportion of Sequenced Samples in RELAB Network, France, Auvergne Rhône Alpes region, September 2024 to January 2025.

The figure shows the weekly number of positive samples tested (stacked bars) and the proportion of sequenced samples (black line) reported through the RELAB network in the Auvergne Rhône Alpes region, France, between weeks 36/2024 and 09/2025. The total number of samples is stratified by sequencing status: sequenced (dark blue) and not sequenced (light blue).

Supplementary Figure S2. Monthly proportion of influenza subtypes among sequenced cases in Auvergne–Rhône–Alpes, November 2024 to February 2025.

Loess-smoothed monthly proportions (span = 0.95) of influenza B/Victoria, A(H1N1)pdm09, and A(H3N2) among sequenced samples from two surveillance sources: HCL (hospital-based laboratories; red) and RELAB-ARA (community laboratories; blue). Shaded areas represent 95% confidence intervals. The three panels show subtype-specific temporal trends from November 2024 to February 2025.

Supplementary Figure S3. Distribution of influenza subtypes by age group in hospital (HCL) and community (RELAB-ARA) surveillance, Auvergne–Rhône–Alpes, 2024–2025 season.

Stacked bar plots showing the relative proportion of influenza A(H1N1)pdm09, A(H3N2), and B/Victoria among sequenced cases across four age groups (<2, 2–17, 18–64, and ≥65 years). Results are presented separately for samples from hospital-based laboratories (HCL) and community-based laboratories (RELAB-ARA). Total numbers of sequenced samples within each age group are indicated above the corresponding bars.

Supplementary Figure S4. Monthly distribution of influenza subtypes in hospital (HCL) and community (RELAB-ARA) surveillance, Auvergne–Rhône–Alpes, 2024–2025 season.

Stacked bar plots showing the proportional distribution of influenza A(H1N1)pdm09, A(H3N2), and B/Victoria among sequenced samples by month from September 2024 to February 2025. Data are presented separately for hospital-based laboratories (HCL) and community-based laboratories (RELAB-ARA). The total number of sequenced samples for each month is indicated above the corresponding bar.

Supplementary Figure S5. Whole-genome maximum-likelihood phylogenetic analysis of pandemic influenza A(H1N1) viruses from ARA, 2024–2025.

Maximum-likelihood tree inferred from *n* = 697 complete A(H1N1)pdm09 genomes collected in the Auvergne–Rhône–Alpes (ARA) region between September 2024 and February 2025. Branch lengths are proportional to the number of nucleotide substitutions per site (scale bar = 0.002). Tip symbols indicate the sequencing laboratory: blue squares, ARA-HCL; red circles, ARA-RELAB-HCL. Filled black circles highlight reference root strains / World Health Organization reference vaccine strains used for tree rooting. Bootstrap values ≥ 80% are shown at the corresponding internal nodes.

The adjacent heat-maps summarise (from left to right): clade assignment, subclade classification, and collection period by month. The heat-maps include all clades and subclades observed; blank cells

correspond to missing annotations. The topology reveals the coexistence of at least two major clades (6B.1A.5a.2a and 6B.1A.5a.2a.1) and multiple subclades (e.g. C.1.9 and D.1) circulating during the study period, with no clear surveillance-system–specific clustering.

Supplementary Figure S6. Phylogenetic tree of influenza B/Victoria viruses (n = 595), Auvergne–Rhône–Alpes, 2024–2025.

Maximum-likelihood tree inferred from 595 complete B/Victoria-lineage genomes generated from influenza retrospective surveillance samples processed by the National Reference Center (NRC) for Respiratory Viruses of the Hospices Civils de Lyon (HCL). The dataset includes samples from hospitalized patients and from the RELAB network, a group of community-based laboratories in the Auvergne–Rhône–Alpes (ARA) region, collected between September 2024 and February 2025. Branch lengths are scaled to the number of nucleotide substitutions per site.

(scale bar = 0.002). Tip symbols indicate the sequencing laboratory: blue squares, ARA-HCL; red circles, ARA-RELAB-HCL. Filled black circles highlight World Health Organization reference/vaccine strains used for tree rooting. Bootstrap values ≥ 80% are shown at the corresponding internal nodes.

The adjacent heat-maps summarise (from left to right): clade assignment, subclade classification, and collection period by month. The topology highlights the diversification of subclades C.3 and C.5 within the V1A.3a.2 background, with no surveillance-system–specific clustering.

Supplementary Figure S7. Whole-genome maximum-likelihood phylogenetic analysis of A(H3N2) viruses, Auvergne–Rhône–Alpes, 2024–2025.

Maximum-likelihood tree inferred from 988 complete A(H3N2) genomes generated from influenza retrospective surveillance samples collected between September 2024 and February 2025. Branch lengths reflect the number of nucleotide substitutions per site. The tree was rooted using WHO reference/vaccine strains. Amino-acid changes associated with the J.2.2-B cluster were identified by parsimony. Seven amino-acid changes were associated with J.2.2-B: five in HA, one in NA, and one in PB2. Three HA mutations, G62R, S145N, and P239Q, were strongly correlated with J.2.2-B.

Supplementary Figure S8. Sensitivity analysis excluding emergency department cases: association between influenza subtype/subclade and hospitalisation, Auvergne–Rhône–Alpes, 2024–2025.

Forest plot of odds ratios (ORs) and 95% confidence intervals (CIs) for hospitalisation according to influenza subtype and subclade. This sensitivity analysis was restricted to hospitalised inpatient cases and excluded emergency department cases. Panel A shows subtype-level analyses and Panel B shows subclade-level analyses. Red points and lines represent univariate logistic regression models and blue points and lines represent multivariable logistic regression models adjusted for age group (reference: 18–64 years) and month of diagnosis (reference: September 2024). The vertical dashed line indicates OR = 1 (no association with hospitalisation). Open circles represent the reference categories within each grouping, fixed at OR = 1 (H1N1 among subtypes, C.1.9.3 among A(H1N1)pdm09 subclades, and J.2.a among A(H3N2) subclades). Filled circles indicate estimated ORs for each subtype or subclade. H1N1 and H3N2 subclades are displayed separately.

Supplementary Figure S9. Weekly number of influenza A(H3N2) sequences overall and for subclade J.2.2-B in hospital (HCL) and community (RELAB-ARA) surveillance, Auvergne–Rhône–Alpes, 2024–2025.

Bar plots show, by ISO week, the number of sequenced influenza A(H3N2) viruses detected through hospital-based laboratories (HCL) and the RELAB-ARA community laboratory network (labelled "RELAB" in the figure). The upper panel displays weekly counts of all A(H3N2) sequences, while the lower panel displays the subset of sequences assigned to subclade J.2.2-B. The x-axis indicates ISO week from 2024-W45 to 2025-W05, and the y-axis shows the number of sequences per week for each surveillance source.

Supplementary Figure S10. Weekly number of influenza A(H3N2) and J.2.2-B subclade sequences by age group in hospital (HCL) and community (RELAB-ARA) surveillance, Auvergne–Rhône–Alpes, November 2024 to January 2025.

Line plots show weekly counts of sequenced influenza A(H3N2) viruses (top panels) and the subset belonging to subclade J.2.2-B (bottom panels), stratified by age group (<2, 2–17, 18–64, 82 ≥65 years). Counts are displayed separately for hospital-based laboratories (HCL; red) and community-based laboratories (RELAB-ARA; blue). The x-axis indicates ISO epidemiological week from 2024-W45 to 2025-W05, and the y-axis shows the number of sequences per week within each age group and surveillance source.

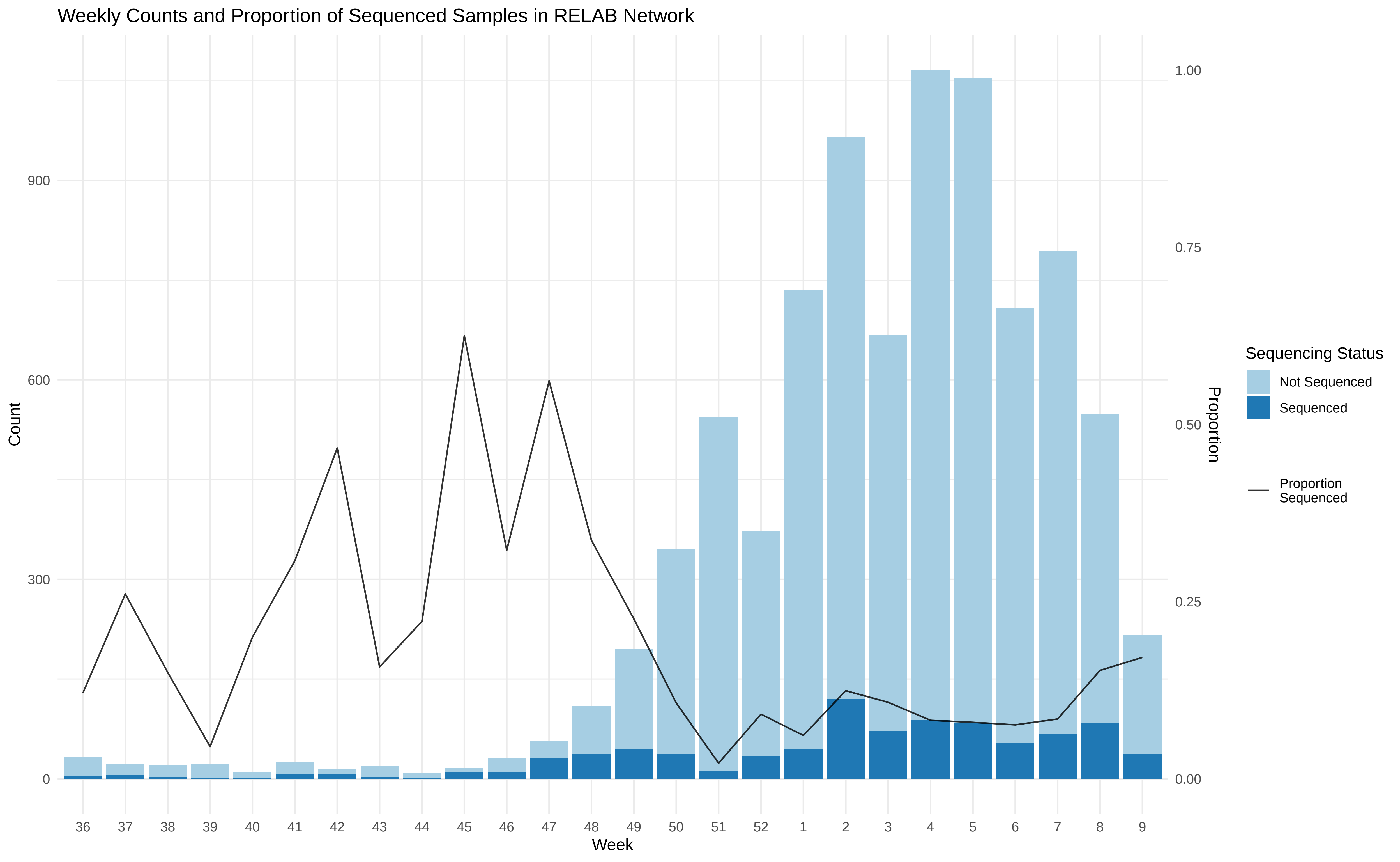
Weekly Counts and Proportion of Sequenced Samples in RELAB Network
Count
900
600
300
0
Proportion
1.00
0.75
0.50
0.25
0.00
Week
36
37
38
39
40
41
42
43
44
45
46
47
48
49
50
51
52
1
2
3
4
5
6
7
8
9
Sequencing Status
Not Sequenced
Sequenced
Proportion Sequenced

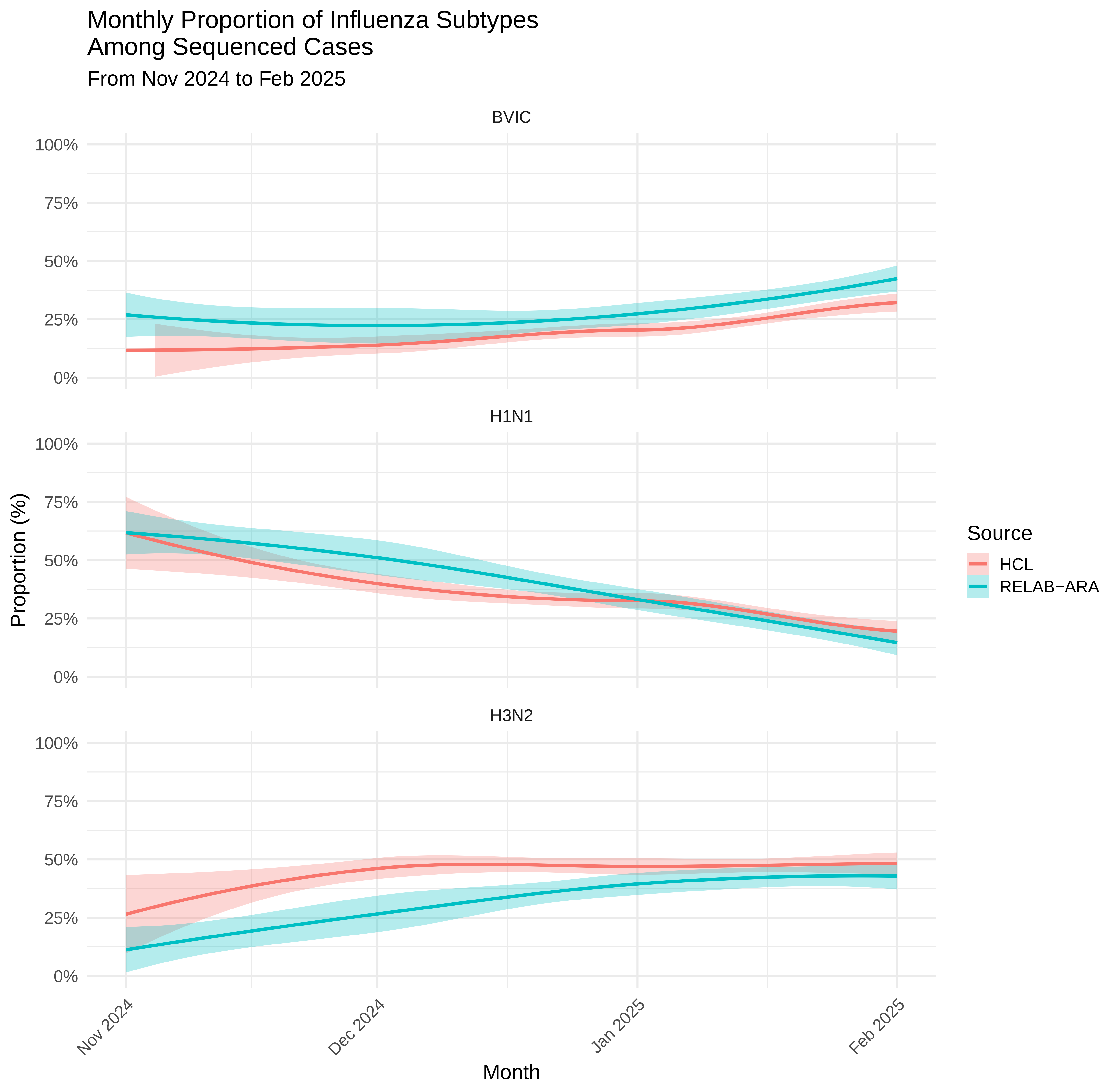

Loess−smoothed trend (span=0.95) with shaded 95% CI

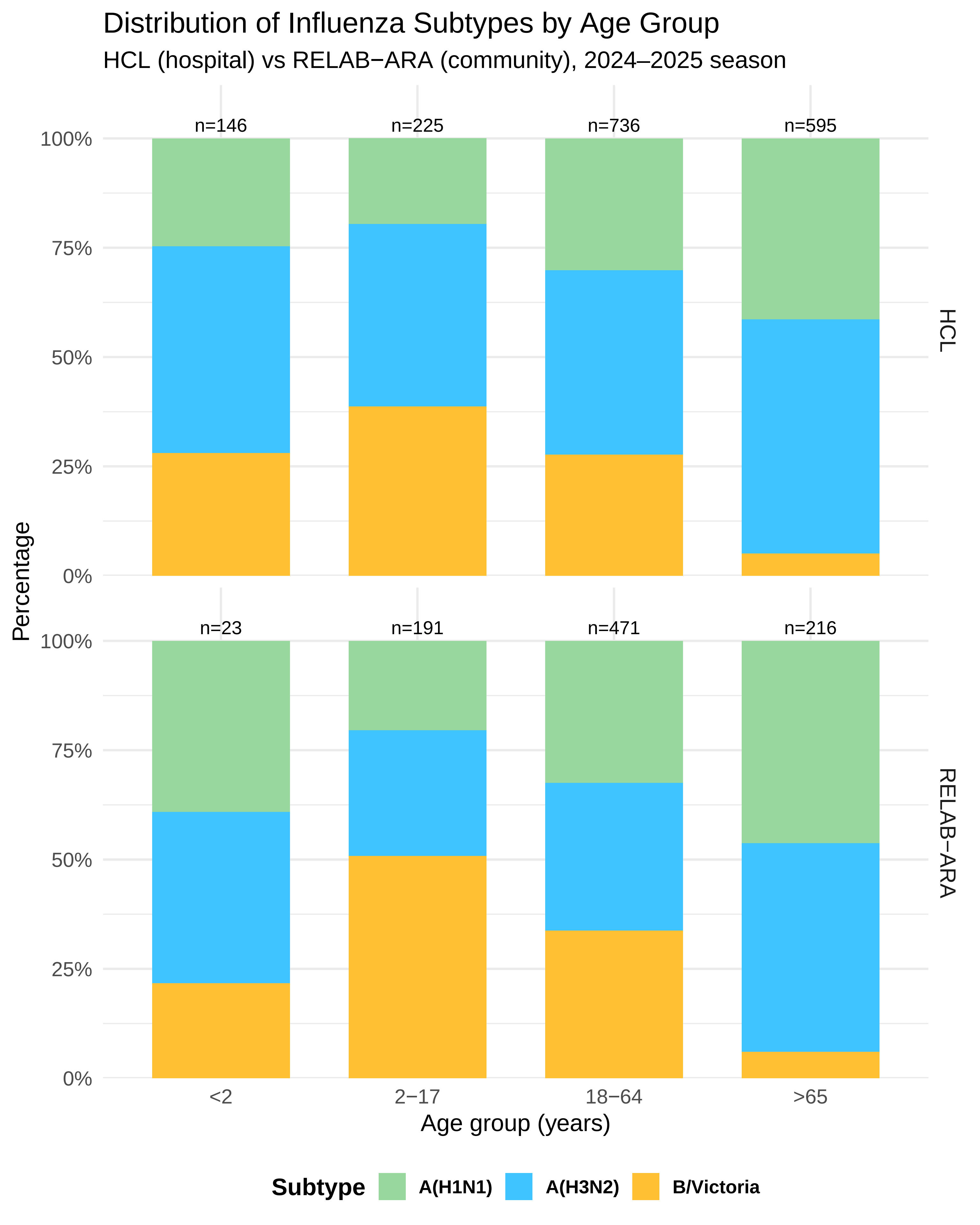

Distribution of Influenza Subtypes by Age Group
HCL (hospital) vs RELAB−ARA (community), 2024–2025 season
n=146
n=225
n=736
n=595
n=23
n=191
n=471
n=216
100%
75%
50%
25%
0%
HCL
RELAB−ARA
Percentage
<2
2−17
18−64
>65
Age group (years)
Subtype
A(H1N1)
A(H3N2)
B/Victoria

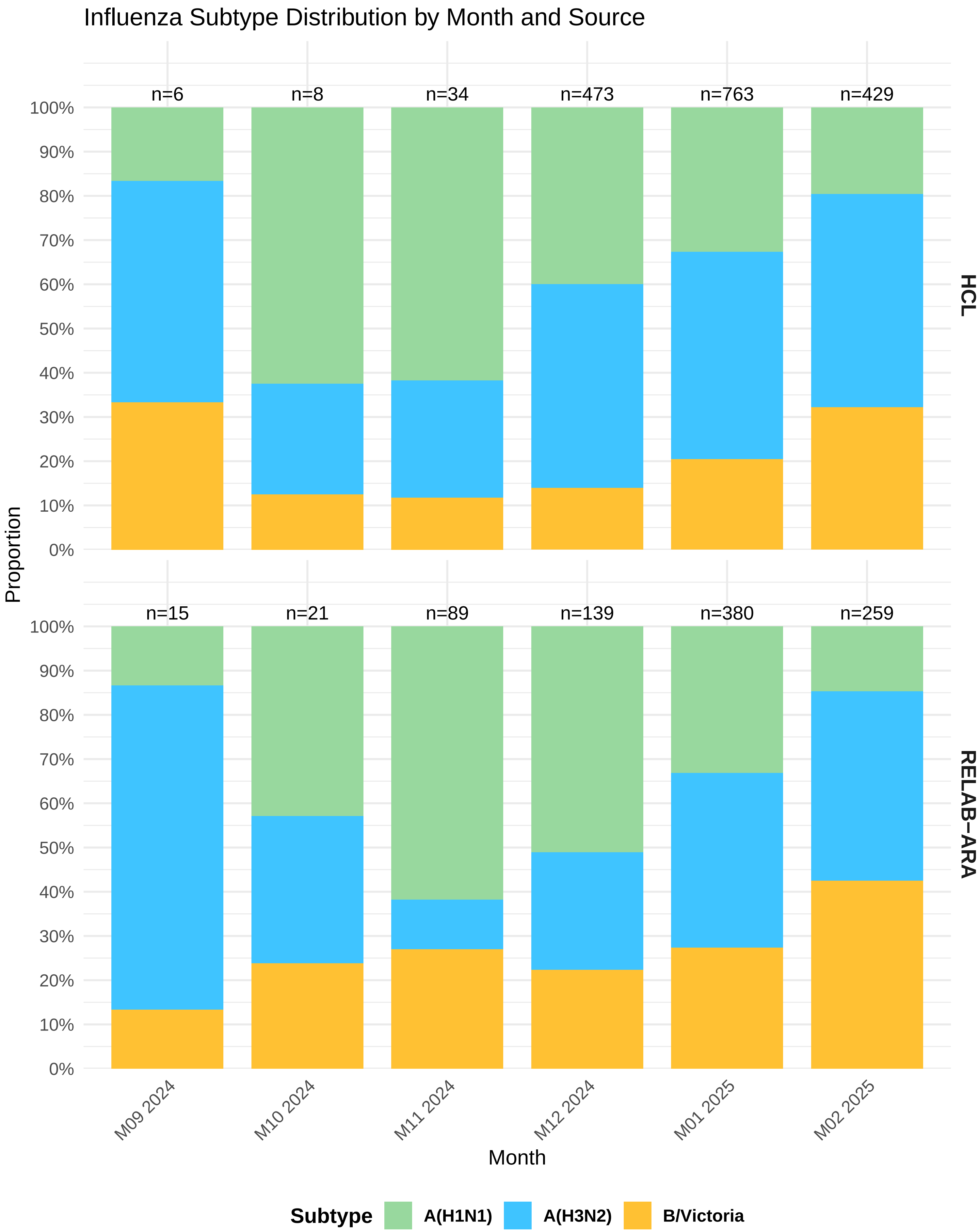
Influenza Subtype Distribution by Month and Source
HCL
RELAB–ARA
n=6
n=8
n=34
n=473
n=763
n=429
n=15
n=21
n=89
n=139
n=380
n=259
Proportion
0%
10%
20%
30%
40%
50%
60%
70%
80%
90%
100%
M09 2024
M10 2024
M11 2024
M12 2024
M01 2025
M02 2025
Month
Subtype
A(H1N1)
A(H3N2)
B/Victoria

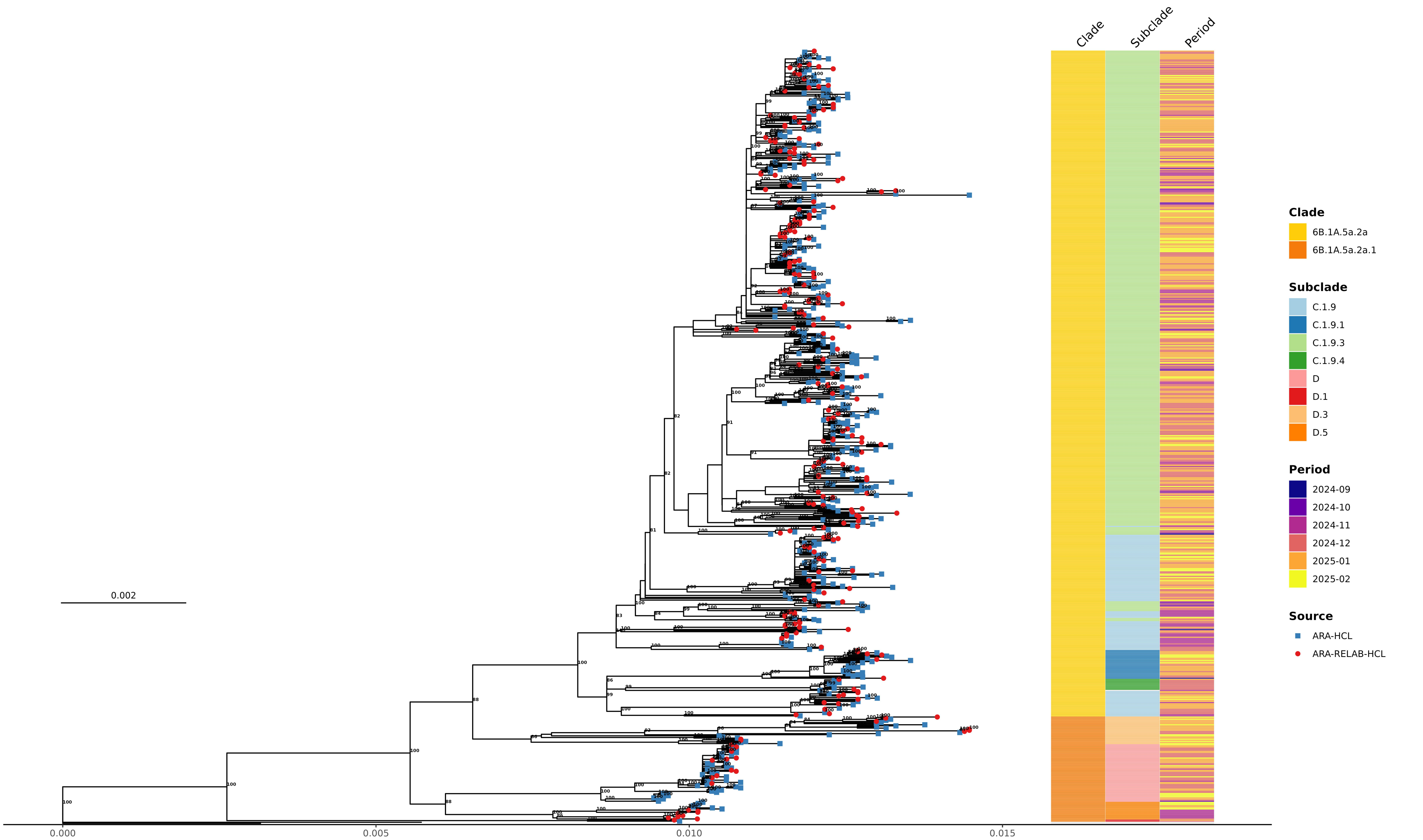
Clade
Subclade
Period
0.002
0.000
0.005
0.010
0.015
Clade
6B.1A.5a.2a
6B.1A.5a.2a.1
Subclade
C.1.9
C.1.9.1
C.1.9.3
C.1.9.4
D
D.1
D.3
D.5
Period
2024-09
2024-10
2024-11
2024-12
2025-01
2025-02
Source
ARA-HCL
ARA-RELAB-HCL

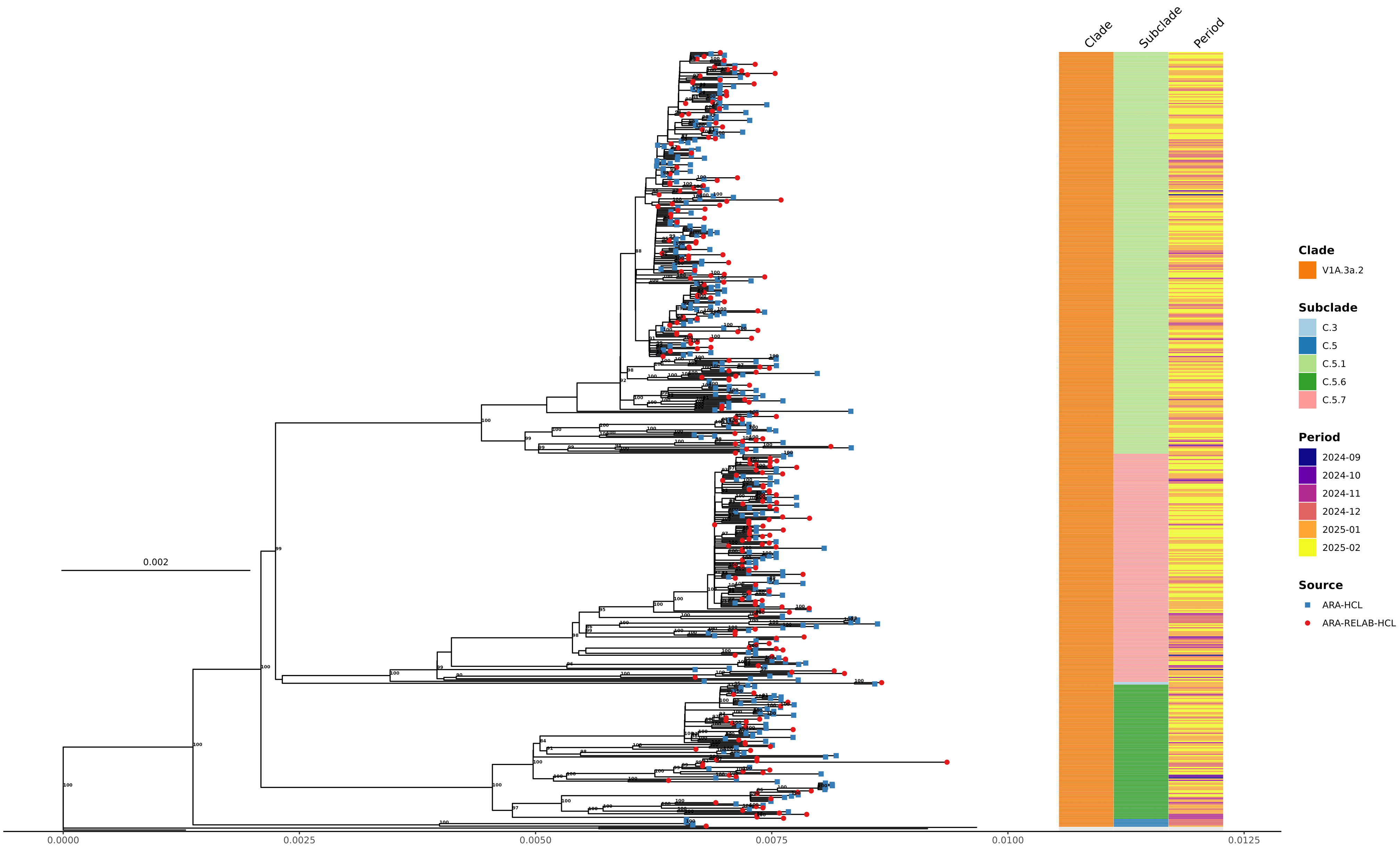

Clade
Subclade
Period
0.002
0.0000
0.0025
0.0050
0.0075
0.0100
0.0125
Clade
V1A.3a.2
Subclade
C.3
C.5
C.5.1
C.5.6
C.5.7
Period
2024-09
2024-10
2024-11
2024-12
2025-01
2025-02
Source
ARA-HCL
ARA-RELAB-HCL

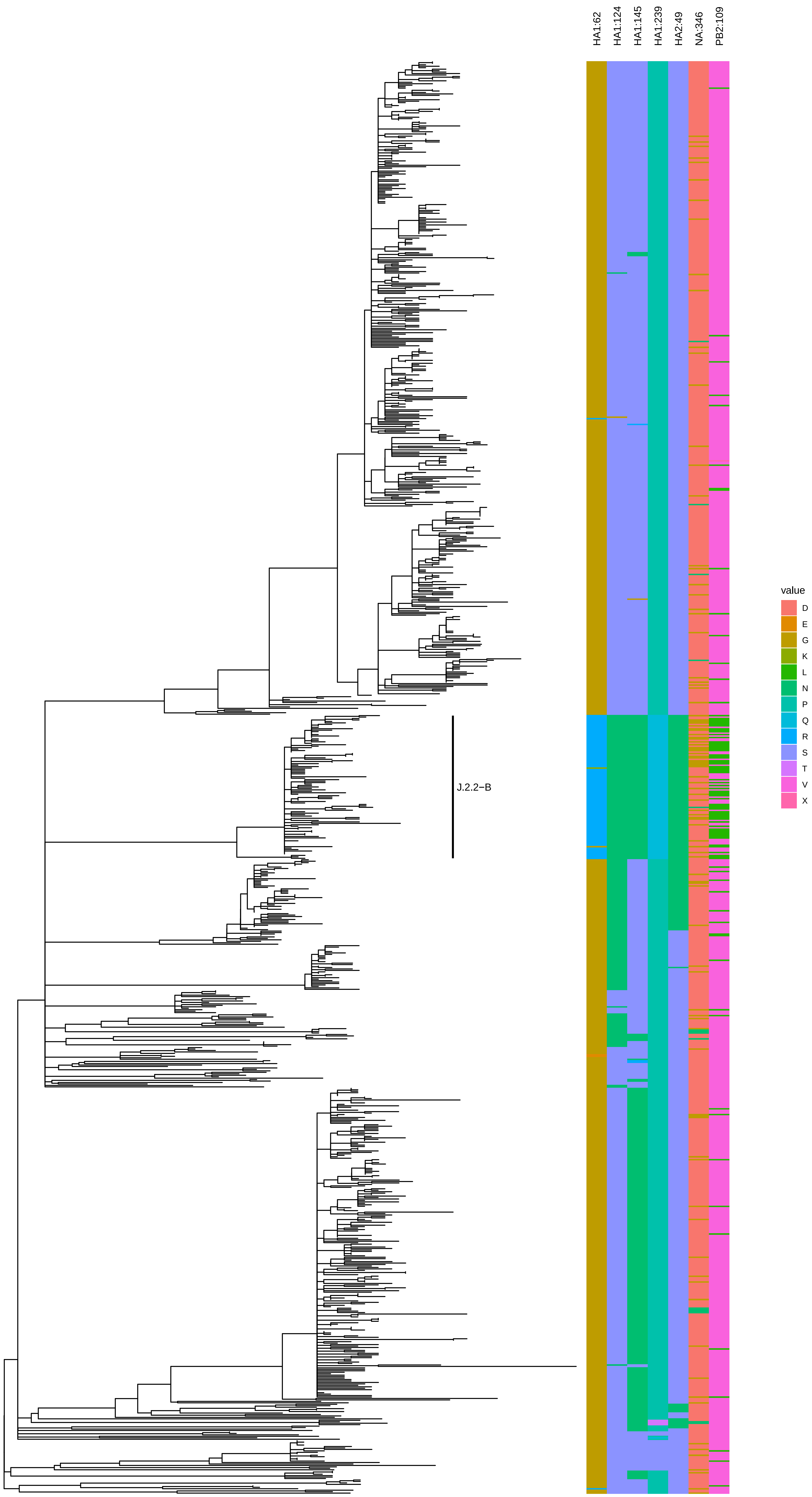

HA1:62
HA1:124
HA1:145
HA1:239
HA2:49
NA:346
PB2:109
J.2.2−B
value
D
E
G
K
L
N
P
Q
R
S
T
V
X

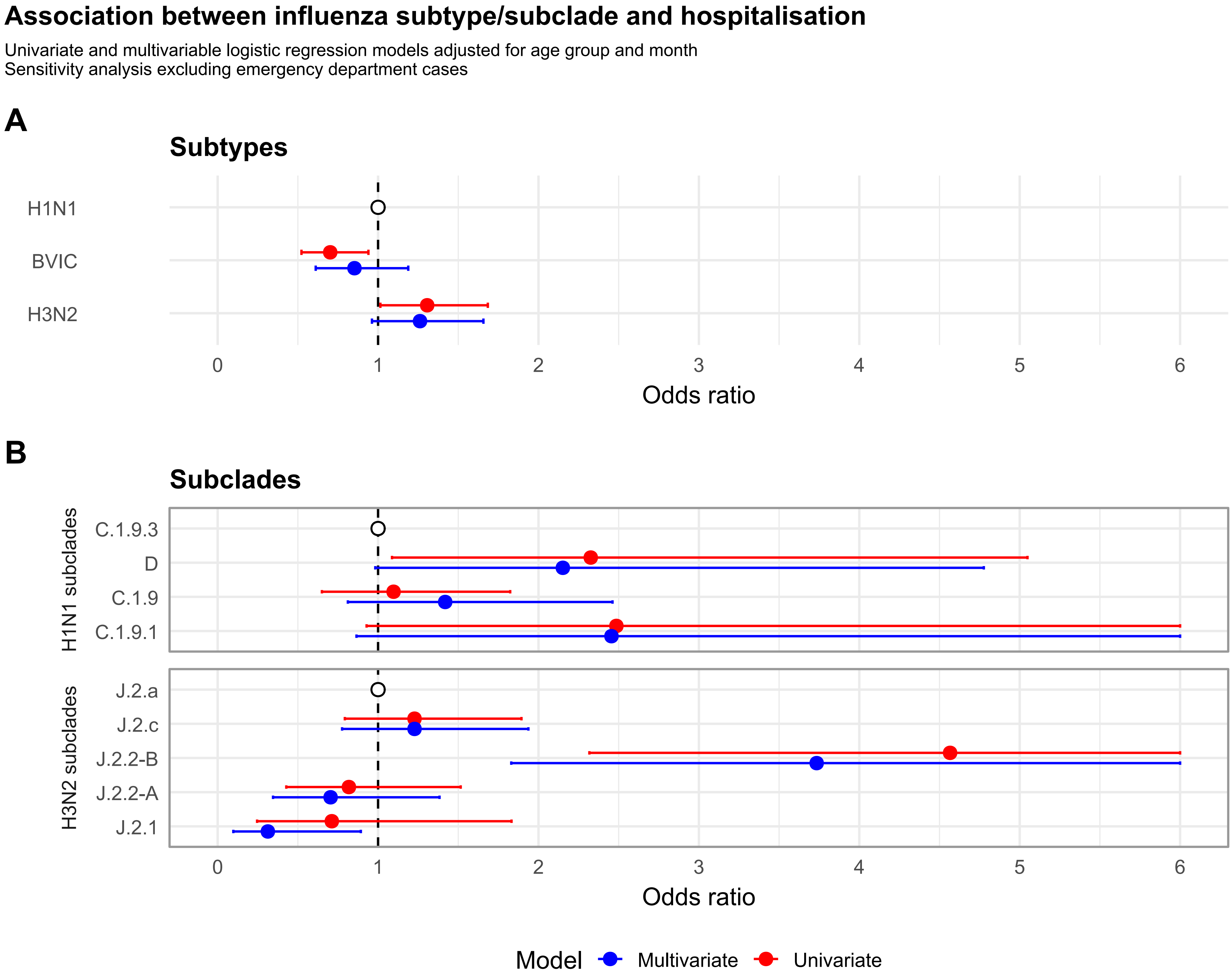
Association between influenza subtype/subclade and hospitalisation
Univariate and multivariable logistic regression models adjusted for age group and month
Sensitivity analysis excluding emergency department cases
A
Subtypes
H1N1
BVIC
H3N2
0
1
2
3
4
5
6
Odds ratio
B
Subclades
H1N1 subclades
C.1.9.3
D
C.1.9
C.1.9.1
H3N2 subclades
J.2.a
J.2.c
J.2.2-B
J.2.2-A
J.2.1
0
1
2
3
4
5
6
Odds ratio
Model
Multivariate
Univariate

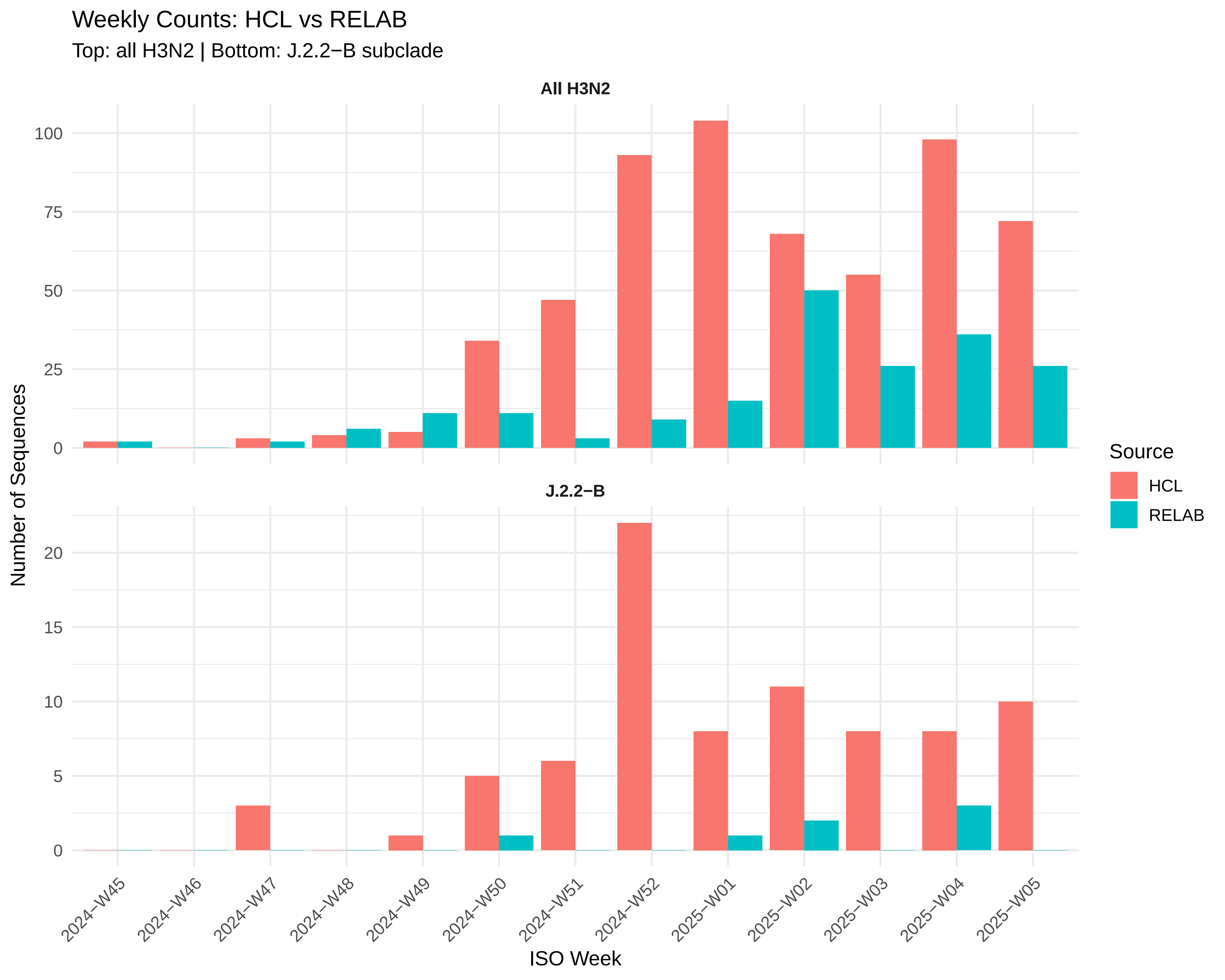

Weekly Counts: HCL vs RELAB
Top: all H3N2 | Bottom: J.2.2−B subclade
All H3N2
J.2.2−B
Number of Sequences
ISO Week
0
25
50
75
100
0
5
10
15
20
2024−W45
2024−W46
2024−W47
2024−W48
2024−W49
2024−W50
2024−W51
2024−W52
2025−W01
2025−W02
2025−W03
2025−W04
2025−W05
Source
HCL
RELAB

Weekly H3N2 vs J.2.2−B by Age Group

Nov 2024–Jan 2025 (HCL vs RELAB)

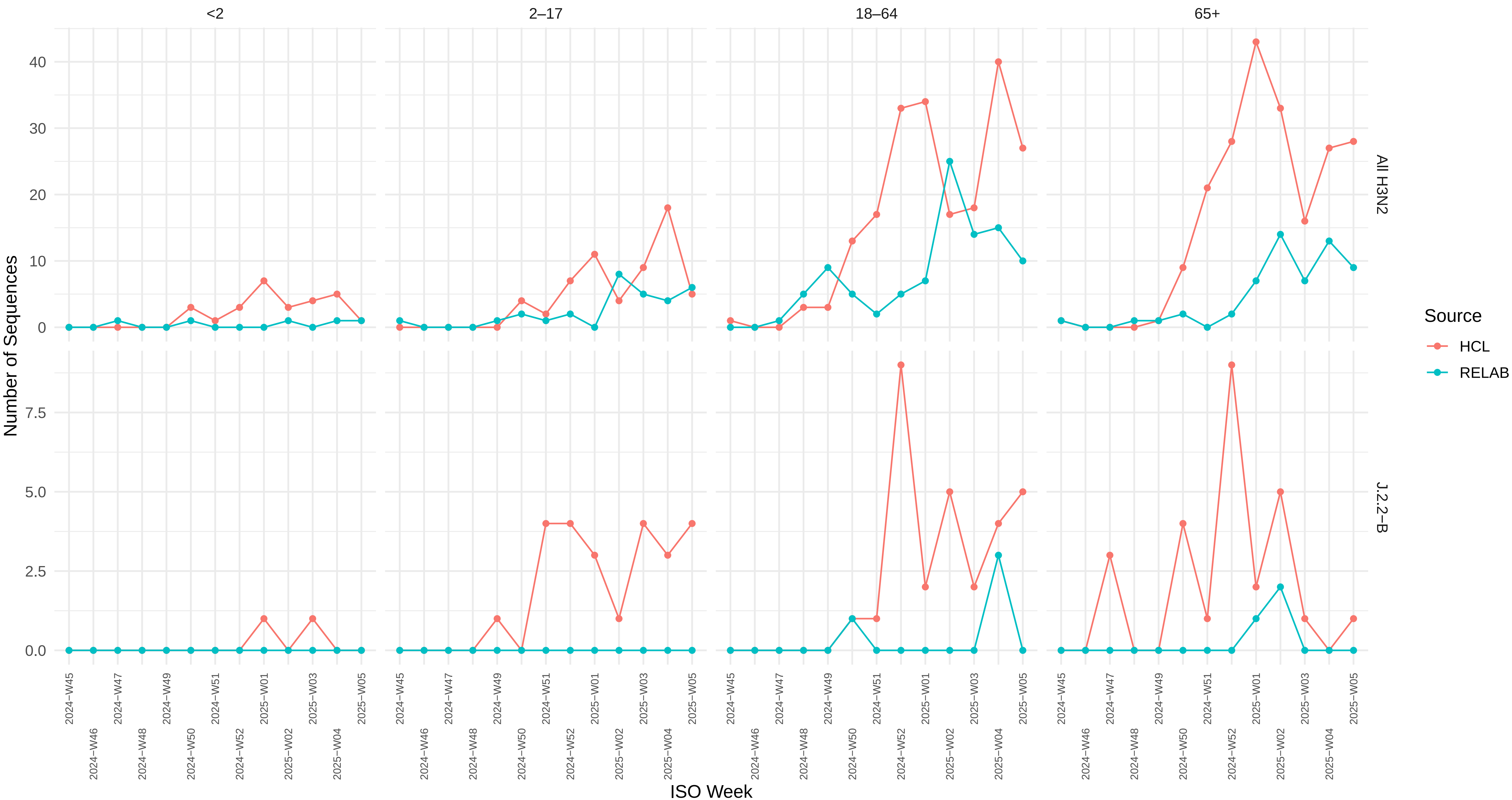

Supplementary Table I Bioinformatics workflow for influenza sequencing data analysis

	Software	version
Trimming of paired reads to remove adapters and low-quality ends, keeping reads >30 bp	cutadapt	
Alignment to reference genomes (pH1N1, H3N2, BVIC)	bwa-mem	
Removal of duplicate reads	picard	
Realignment to improve indel detection	abra2	
Clipping of read ends containing primer sequences	samtools ampliconclip	
Variant calling for variants ≥5% frequency	bcftools	
Decomposition and normalization of variants	vt	
Filtering variants to eliminate false positives	bcftools	
Reporting of consensus variants ≥50% frequency	custom script	
Interpretation of positions with depth ≥10X for final consensus	custom script	

Clade assignment of sequences	nextclade<br>(CLI)
