# Supplementary material for: Emergence of a genetically distinct cluster of influenza A(H3N2) viruses within subclade J.2.2 associated with hospitalization during the 2024–2025 season in Auvergne–Rhône–Alpes, France
Source: Microb Genom. 2026 Sep 22;12(9):001810. doi: 10.1099/mgen.0.001810 (PMC13597097; doi:10.1099/mgen.0.001810)
Supplement: Supplementary file 2 [file mgen-12-01810-s002.pdf]

Supplementary Appendix

All genome sequences and associated metadata supporting the findings of this study can be accessed through the persistent digital object identifier https://doi.org/10.55876/gis8.260619vs

In addition to the minted DOI, GISAID also communicates the aggregation of GISAID accession numbers (EPI_ISL_IDs) through the corresponding EPI_SET_260619vs identifier to facilitate both, the acknowledgment of all data contributors and the direct retrieval of the underlying data from GISAID used in this study.

Influenza Virus Data Summary

GISAID Identifier	Digital Object Identifier	Number of individual viruses	Data Collection range	Number of countries/territories
EPI_SET_260619vs	https://doi.org/10.55876/gis8.260619vs	2,615	2024-09-02 to 2025-02-28	1
